# Formaldehyde, Oxidative Stress, and FeNO in Traffic Police Officers Working in Two Cities of Northern Italy

**DOI:** 10.3390/ijerph17051655

**Published:** 2020-03-04

**Authors:** Giulia Squillacioti, Valeria Bellisario, Amelia Grosso, Federica Ghelli, Pavilio Piccioni, Elena Grignani, Angelo Corsico, Roberto Bono

**Affiliations:** 1 Department of Public Health and Pediatrics, University of Torino, 10121 Torino, Italy; giulia.squillacioti@unito.it (G.S.); valeria.bellisario@unito.it (V.B.); federica.ghelli@unito.it (F.G.); 2 Division of Respiratory Diseases, S. Matteo Foundation–University of Pavia, 5001 Pavia, Italy; amelia.grosso@gmail.com (A.G.); angelo.corsico@unipv.it (A.C.); 3 Unit of Respiratory Medicine, National Health Service, ASL TO2, 10121 Torino, Italy; papiccioni@gmail.com; 4 Maugeri Scientific Clinical Institutes, 5001 Pavia, Italy; elena.grignani@icsmaugeri.it

**Keywords:** public health, traffic police officers, formaldehyde, 15-F2t-isoprostane, FeNO

## Abstract

Personal air formaldehyde (air-FA) was measured as risk factor of airways inflammation and oxidative stress (SO) induction. Overall, 154 police officers were enrolled from two differently urbanised Italian cities, Turin and Pavia. Urinary F2t-isoprostane (15-F2t-IsoP), a prostaglandin-like compound, was quantified as a biomarker of general OS in vivo and fractional exhaled nitric oxide (FeNO) was measured for monitoring local inflammatory processes. Urinary cotinine was quantified as a biomarker of tobacco smoking exposure. Traffic police officers living in Turin showed an increased level of log air-FA (*p* < 0.001), equal to +53.6% (*p* < 0.001). Log air-(FA) mean values were 3.38 (C.I. 95% 3.33–3.43) and 2.84 (C.I. 95% 2.77–2.92) in Turin and Pavia, respectively. Log (air-FA) was higher in “outdoor workers” (3.18, C.I. 95% 3.13–3.24, *p* = 0.035) compared to “indoor workers”, showing an increase of +9.3%, even controlling for sex and city. The analyses on 15-F2t-IsoP and FeNO, both adjusted for log air-FA, highlighted that OS and inflammation were higher (+66.8%, *p* < 0.001 and +75%, *p* < 0.001, respectively) in Turin traffic police officers compared to those from Pavia. Our findings suggest that even low exposures to traffic-related emissions and urbanisation may influence both general oxidative stress levels and local inflammation.

## 1. Introduction

Due to their toxicological and/or carcinogenic properties, some volatile organic compounds (VOCs), aromatic hydrocarbons and carbonyls have increasingly gained attention in the international scientific community dealing with public health issues [1,2,3]. In the last decades, researchers, interested in non-communicable diseases and prevention, focused on the interaction between the aforementioned chemicals and human health, especially in preventive terms [4,5,6]. Formaldehyde (FA), the simplest among the aldehydes, is an ubiquitous pollutant in living and occupational environments [7].

FA may directly originate from natural sources, from several anthropogenic activities and from indirect product via the photochemical oxidations of hydrocarbons in the atmosphere [8,9]. FA has been detected in several occupational settings involving wood-based materials, laminates, paints and urea-based resins [10] and is used as disinfectant and preservative in hospitals and in pathology units [11]. According to the International Agency for Research on Cancer (IARC) [12] and the United States Environmental Protection Agency (US EPA) [13], FA is classified as a human carcinogen. FA-related exposures are connected to many other health effects, such as eye and respiratory tract irritations, allergic contact dermatitis and bronchial asthma [12,14].

Some previous studies investigated the influence of air pollution and FA on inflammation and Oxidative Stress (OS) induction [11,15,16] and on DNA toxicity [17,18]. Conversely, data on personal exposure to FA and OS induction in urban air settings are still lacking. Exposure to environmental pollution may lead to OS and inflammation, which in turn are able to determine biological outcomes and health impairments [19]. Exposure to FA may enhance oxidant species and impair the antioxidant system leading to OS. This latter can result in structural and enzymatic changes in organs [20], cytotoxic effects [21] and carcinogenic processes.

Raffic police officers may undergo long-term exposures to traffic-related pollutants [17,18]. For this reason, they can represent a population model suitable to describe some potential risky conditions for health in urban settings. Therefore, this population was considered suitable to assess the exposition to outdoor and indoor FA and some potentially consequent health effects such as OS and airway inflammation.

Although air-FA is usually higher indoors, the exposure in outdoor environments may considerably contribute to the human daily intakes of FA, the concentration of which is extremely variable in relation to many aspects such as the demographic density, the urban conformation and traffic management, the characteristics and the dimension of the human activities. In this respect, we speculated that the more urbanised cities may be considered greater sources of air-FA, representing a potential additional risky condition for health, compared to smaller cities.

This cross-sectional study aims to evaluate the role of FA exposure and the urbanisation level on OS induction, quantified by urinary 15-F2t-Isoprostane (15-F2t-IsoP), and on airway inflammation, measured by the fraction of exhaled nitric oxide (FeNO). It is notable that 15-F2t-IsoP is a reliable and sensitive biomarker of OS, used in several other epidemiological studies [10,11,16]. FeNO is measured in a non-invasive way and is a biomarker of eosinophilic airway inflammation, useful in monitoring inflammatory processes due to air pollution exposure [20].

A sample of traffic police officers from Turin and Pavia, two north-western Italian cities with different levels of urbanisation, was enrolled.

## 2. Materials and Methods

**Figure 1:**
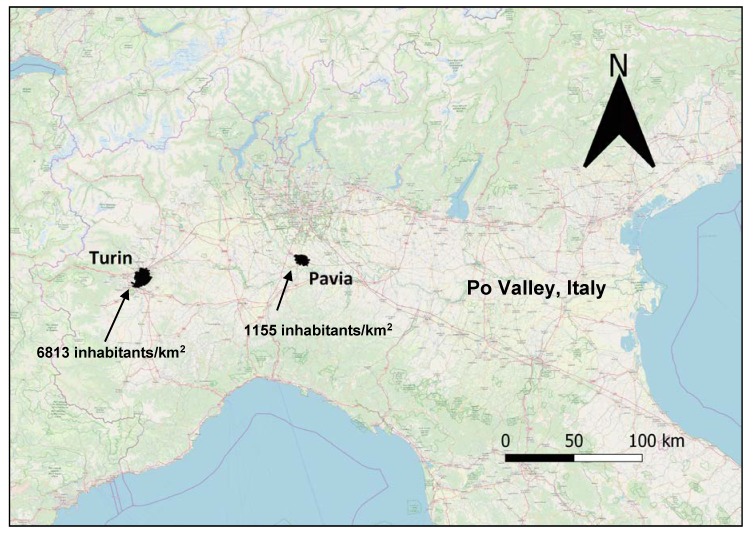
Map showing study area. The human density of the two cities, about 160 km apart, is reported as inhabitants per square kilometer.

In total, 154 traffic police officers were enrolled in this study from November 2015 to March 2016; 109 of them were from Turin and 45 from a smaller town, Pavia. Turin (886,837 inhabitants) is located 239 m above sea level (a.s.l.), and has a population density per km^2^ equal to 6813, while Pavia (72,612 inhabitants) is located 77 a.s.l. and has a population density equal to 1155 per km^2^. Both cities are located in the north-western part of Italy, in the Po Valley (Figure 1), and are approximately 160 km from each other. The Po Valley (470,000 km^2^) is one of the largest European plains, and is relatively homogeneous in terms of lifestyle, social and working conditions. In ecological terms, its climate is continental and, due to frequent thermal inversion episodes, vertical and horizontal air exchanges are more difficult compared to other areas of Europe, thus the air quality is poor [22].

The workers eligible to participate in the study were those working as traffic police officers in the urban areas of Turin and Pavia, completing different outdoor tasks such as traffic management (for a maximum of 2 h per working shift) as well as those working indoors. The invitation to participate was addressed to workers of both sexes and no other exclusion criteria were considered. In total, 154 traffic police officers participated by providing their written informed consent. The epidemiological sample did not involve the enrolment of subjects as controls. This is because the purpose of the study, as previously mentioned, was to compare two urban realities and different tasks, performed indoors and outdoors, without focusing on exposed and unexposed subjects.

On the day set for sampling, the subjects wore during the working shift (8 h) a personal air-sampler (Radiello^®^) to measure air-FA [11] (https://www.restek.com/pdfs/radiello-manual.pdf). At the end of the working shift, the traffic police officers filled out a questionnaire and provided a spot of urine for the quantification of 15-F2t-IsoP and cotinine, as biomarkers of OS and tobacco smoking exposure, respectively. Moreover, two groups of pulmonologists measured, at individual level, FeNO as a marker of airway eosinophils inflammation.

This study obtained ethical approval in accordance with the Helsinki Declaration of 1975 (“Fondazione I.R.C.C.S. Policlinico San Matteo, Pavia” protocol number: 20130000718).

### 2.1. Questionnaire

The same skilled person administered the questionnaire to each subject in the Turin and Pavia headquarters of the traffic police officers, collecting details about anthropometric characteristics, job tasks and location, years of service and potential confounders in OS induction such as diet, physical activity and tobacco smoke habits.

### 2.2. Personal Air-FA

Air-FA samples were collected for a whole working shift (8 h), using passive personal air samplers working with the radial symmetry (Radiello^®^), clipped near the breathing zone of the subject. The personal air samplers were equipped with a specific sorbent tube containing a 35–50 florisil mesh coated with 2,4-dinitrophenylhydrazine (DNPH). DNPH reacts with FA yielding 2,4-dinitrophenylhydrazone, which was subsequently quantified by high performance liquid chromatography (HPLC) according to the NIOSH method No. 2016 [23]. Briefly: the sampling rate value Q for Radiello^®^ at 298 K (25 °C) and 1013 hPa is 99, the linearity range in μg/m^3^ is 1000 ÷ 4,000,000, the limit of quantitation is μg/m^3^ = 0.1, and uncertainty is at 2σ%, is 13.8.

The following materials are required to proceed with desorption: HPLC or spectroscopy grade acetonitrile, class A volumetric pipette, capacity 2 mL, micropore filter membranes, porosity 0.45 μm, solvent resistant. Procedure: 2 mL acetonitrile were introduced directly in the cartridge tube, recap and stir from time to time for 30 min. The resulting solution was filtered and kept well capped until analysis time. When the analysis was delayed, the solution was stored at 4 °C. Materials for instrumental analysis: reverse phase C18 HPLC column, length 150 mm, 4.6 mm diameter, 5 μm packing particle size. The HPLC apparatus was capable of elution gradient and UV detection. Procedure: the detector was set at a wavelength of 365 nm. Between 10 and 50 μL of the solution was injected and eluted at flow of 1.9 mL·min−1. An isocratic elution was done with acetonitrile/water 38:62 *v*/*v* for 10 min, up to acetonitrile/water 75:25 *v*/*v* in 10 min, and reverse gradient to acetonitrile/water 38:62 *v*/*v* in 5 min. Finally, the detection limit was calculated as the sample concentration, providing a signal-to-noise ratio of 3. The quantification limit was considered to be twice when compared to the detection limit: 0.10 μg mL^−1^ and 0.05 μg mL^−1^ respectively. The CV values were <5%.

### 2.3. Biological Analyses

Urinary samples were collected at the end of the working shift, divided into aliquots, and stored at −80 °C until analyses. In order to normalise the individual excretion rate of all biological parameters, the first aliquot was used to quantify the urinary creatinine concentration by the Kinetic Jaffé method [24]. Since active and passive tobacco smoke exposure may exert its pro-oxidant effect acting as a potential confounder, urinary cotinine was measure in another aliquot, using a specific ELISA kit (Abnova Corporation, Jhongli, Taiwan) following the manufacturer’s instructions. Finally, the 15-F2t-IsoP was quantified by the ELISA technique following the manufacturer’s instructions [16].

### 2.4. FeNO Measurements

FeNO was measured in accordance with the American Thoracic Society and European Respiratory Society recommendations [25], by the CLD 88 chemiluminescence analyser (Eco Medics, Durnten, Switzerland), at an exhaled flow of 50mL/s and given in part per billion (instrument limit values ranging between 0.1–5000 ppb). The same group of pulmonologists performed the measurements in the Turin and Pavia traffic police officer headquarters. Participants were requested to refrain from smoking, eating, drinking and doing strenuous exercise for one hour prior to the measurement. The instrument was turned on at least 15 min prior to use and set for a 10 s inhalation. Each subject underwent measurement after receiving detailed explanations by the personnel. A nearby mirror helped participants in exhaling at the correct speed. All subjects were in sitting position and were asked to empty their lungs through a single long exhalation and to exhale slowly and steadily into the mouthpiece. If the participants were unable to complete the test at the first attempt, more attempts, no more than nine per person, were repeated and recorded.

### 2.5. Statistical Analysis

Descriptive analyses were carried out with the chi-square test for categorical variables (gender, job duties, cities), and the t-test or Mann–Whitney U-test for quantitative parameters (age, BMI, FeNO, cotinine, 15-F2t-IsoP, and FA), as appropriate. Correlation analyses were performed using the non-parametric Spearman’s test to investigate the correlations between the biomarkers of OS and tobacco smoke exposure (15-F2t-IsoP and cotinine) and of OS and personal air-FA (15-F2t-IsoP and FA). A logarithmic transformation (Log-e) was performed for all the variables that showed a non-normal distribution (15-F2t-IsoP and FA); the normality of the distribution was tested using the Shapiro–Wilk test. Multiple linear regression (MLR) models were used to test the relationship and strength of the association between the dependent variable, independent variable and covariates or confounders (sex, tobacco smoke, sampling location, and job duties, depending on the specific model). MLR is an extension of ordinary least squares (OLS) regression involving more than one explanatory variable. MLR has been used to check the association between dependent and independent variables, mainly based on their approximately linear relationship and on the absence of collinearity among the covariates. The most parsimonious model has been selected, comparing several models that included a different set of variables, selected by a stepwise method. All categorical variables included in the model were dichotomous (0–1).

Moreover, the analysis on predictive margins was performed to deeply investigate the influence of job duties on FA exposure between the two locations, the difference of 15-F2t-IsoP between Turin and Pavia, controlling for personal air-FA and FeNO variation among venues controlling for both FA and cotinine. The level of significance was set at *p* ≤ 0.05 (two-tailed) for all tests. All analyses were performed using STATA SE v14.2 (Stata Corp, College Station, TX, USA).

## 3. Results

Table 1 reports the general details of the epidemiological sampling between the two cities. Although more police officers were sampled in Turin (71%) than in Pavia (29%), no differences were observed in the distribution of age, BMI, gender and job duties (carried out indoors and outdoors) between the two cities. As shown in Table 2, the mean values of urinary 15-F2t-IsoP and FeNO are significantly different across locations (both *p* < 0.001), as well as personal exposure levels of air-FA (*p* < 0.001). Conversely, urinary cotinine shows similar distributions among subjects working in the two sampling locations, highlighting that both considered populations show similar smoking habits.

**Figure 2:**
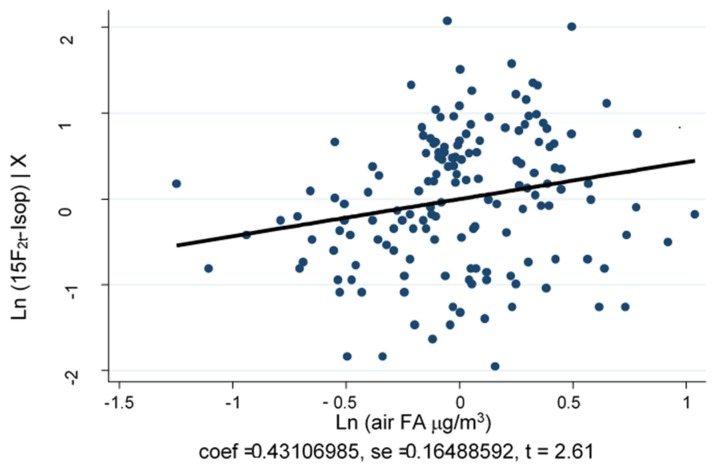
Correlation between Air-formaldehyde (FA) and 15-F_2t_-IsoP. The correlation is significant and positive (Spearman’s rho = 0.241, *p* = 0.003).

**Figure 3:**
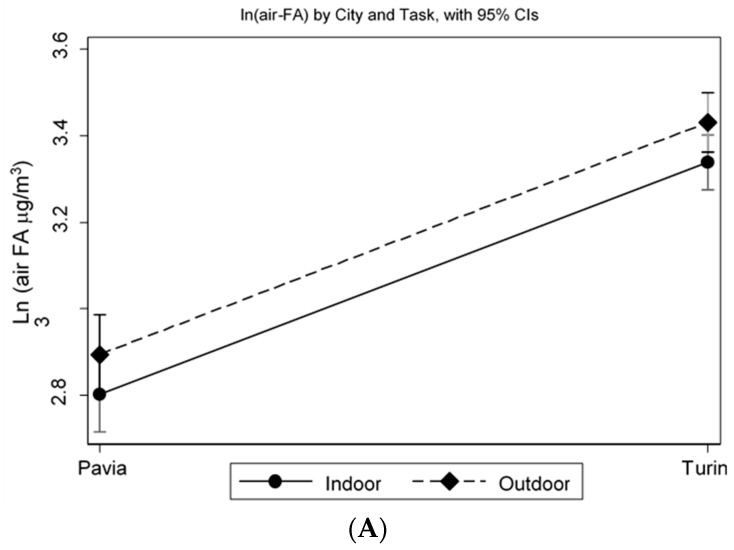

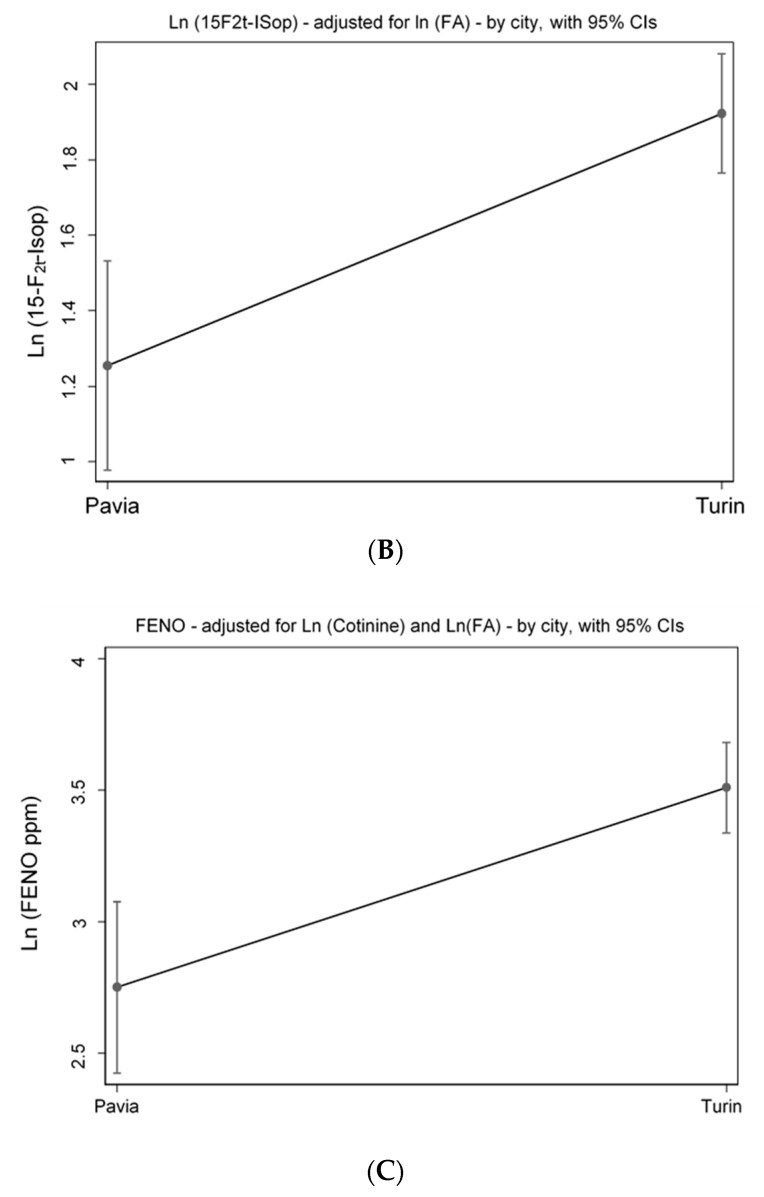
MLR model. Log (air-FA), in Turin and in Pavia, indoors and outdoors (**A**), Log (15 F2t-IsoP) (**B**) and log (FeNO) (**C**).

As depicted in Figure 2, air-FA and 15-F2t-IsoP are significantly and positively correlated (Spearman’s rho = 0.241, *p* = 0.003). The MLR model, adjusted for job duties and sex, shows that traffic police officers working in Turin have a significantly higher level of log (air-FA), 53.6% more than workers in Pavia (*p* < 0.001). In particular, the two log (air-FA) mean values are 3.38 (C.I. 95% 3.33–3.43) in the traffic police officers working in Turin and 2.84 (C.I. 95% 2.77–2.92) in the traffic police officers working in Pavia. Moreover, controlling for sex and city, log (air-FA) is significantly higher in traffic police officers who carried out their job duties mostly outdoors (3.18, C.I. 95% 3.13–3.24, *p* = 0.035), with an increase of 9.3% compared to traffic police officers working indoors (Figure 3A). The analyses of the OS biomarker highlight that, controlling for log (air-FA), the levels of 15-F2t-IsoP remain significantly higher (+66.8%) in traffic police officers working in Turin, compared with those employed in Pavia (*p* < 0.001) (Figure 3B). Furthermore, the MLR model adjusted for the level of cotinine and for air-FA exposure shows that traffic police officers working in Turin have significant higher levels of FeNO (+75%) compared to those working in Pavia (*p* < 0.001) (Figure 3C). Finally, FeNO is negatively correlated with urinary cotinine, thus with tobacco smoke exposure. Although not significantly (*p* = 0.07), this tendency is consistent with previous studies [26,27,28,29].

## 4. Discussion

Air pollution is a matter of concern for human health due to its potential toxicological and/or carcinogenic effects on humans. Long-term exposures to air pollution, although in low doses, are recognised risk factors for the onset or exacerbation of several diseases, including respiratory infections and inflammations, cardiovascular impairments and cancer [30,31,32]. Among airborne VOCs, FA gained attention because of its toxicological and carcinogenic properties and for its primary and secondary origin in urban ambient air [12]. FA levels usually range between 1 and 20 µg/m^3^ in urban air [33,34], with measurements that are usually higher in personal air compared to those measured by fixed samplers. This difference may depend on the complexity of the scenario described by the personal air-samplers. In fact, they are able to quantify a more comprehensive exposure accounting for all locations visited by the subjects.

In indoor environments, sources emitting FA are numerous, and air exchanges and dilutions processes are scarcer than in outdoor environments [8,35,36]. However, this study analysed a specific scenario of exposure, because the traffic police officers spend most of their working shift outdoors, differently from many other workers. Specifically, exposure to air-FA of traffic police officers is significantly higher in Turin than in Pavia, the smaller town. This result may depend on the different level of urbanisation.

Limiting the observation to outdoor pollution, the Po Valley is one of the most polluted areas in Europe. The orographic barriers enclose this area, characterised by weak winds and depressed surface; these factors provide less chances of air dilution processes, keeping the concentrations of air pollution high [37,38,39,40].

As above-mentioned, Turin and Pavia have different population densities and different air-FA concentrations. Besides the general ambient air quality in the two different urban contexts, this result emphasises the importance of traffic-related air-FA exposure. Additionally, the use of personal air-samplers has adequately characterised the individual exposures, allowing deeper investigations about the potential biological effects and health risks for the workers who participated in the study.

The analyses of general OS and local airways inflammation confirm that air pollution, tobacco smoking and air-FA exposures are able to determine biological effects, reflecting the interaction between environment and human exposure. Furthermore, our results show that the OS biomarker is significantly and positively correlated with both air-FA exposures and urinary cotinine (both *p* < 0.001). Interestingly, 15-F2t-IsoP is higher in traffic police officers employed in Turin if compared to those working in Pavia, even controlling for FA exposures. The model adjusted for cotinine, diet, and BMI did not reach statistical significance, suggesting that other factors, not considered in this study, could contribute to determining an increase of 15-F2t-IsoP, which remains aspecific, besides its reliability in assessing OS status. Nevertheless, the modification of the behaviour of isoprostane only for FA appears to be a finding of considerable importance, even if the correlations are weak.

The traffic police officers of the two cities report a FeNO mean value ranging from 25 to 50 ppb. In particular, higher FeNO levels were recorded in the traffic police officers of Turin. Nevertheless, no correlations were found between FeNO and FA, 15-F2t-IsoP and cotinine, respectively. Unlike other studies [41,42], our findings do not show higher FeNO levels in men, nor in older subjects. FeNO levels measured in our study were classified as “intermediate FeNO”, according to ATS [43] guidelines, which state that cautious interpretation is required at these values, considering the clinical contexts and the individual characteristics of the subjects (e.g., age and gender).

Differently from other authors [42], no relation has been found between FeNO and allergies. Finally, adjusting for sampling locations, FeNO shows a negative association with cotinine levels, although not significant (*p* = 0.07). This result is consistent with previous studies [41,44,45], since tobacco smoke is a recognised factor in the downregulation of the nitric oxide synthase, resulting in lower FeNO levels [42]. At this concern, several mechanisms have been proposed to explain the influence of tobacco smoke on FeNO levels. Nitrogen oxide in respiratory tract originates from inducible NO synthase (iNOS), regulated by interferon gamma (IFN-γ). Tobacco smoke may interfere in this pathway by reducing IFN-γ or directly inducing oxidative processes in the airways leading to the scavenging of NO [44].

## 5. Conclusions

In conclusion, our results suggest that even low doses and relatively small differences among environmental air-FA exposures may contribute to OS induction and airways inflammation. Traffic-related emissions and population densities play an important role in the air pollution exposure of workers who carry out their job duties mostly outdoors, as the traffic police officers do. These latter are exposed to a wide range of airborne pollutants, many of which are capable of inducing oxidative stress and airways inflammation.

Personal exposure measurements, quantified by personal air-samplers, are more accurate than those measured by fixed samplers and better represent the air quality, especially in complex environments such as the urban air. In other words, fixed sampling stations of the Regional Agency for the Protection of the Environment (A.R.P.A.) were not able to accurately describe the individual exposure to air pollutants during the working shift. There are five fixed sampling stations in Turin and two in Pavia, while the personal samplers have been worn by each worker (*n* = 154). Finally, FA is not regularly sampled by fixed sampling stations of A.R.P.A.

A limitation of this study is that only the exposure to FA was measured. However, FeNO, OS, tobacco smoke and air-FA were quantified by standardised and reproducible methods; thus, the comparison between two different polluted cities, located within the same geographic area in the south part of Europe, underlines the role of the urbanisation as an environmental human health risk factor. Future perspectives in this sense may drive further analyses such as the “greyness” quantification in urban environment, in order to better understand how the urbanisation interacts with human exposure and the consequent human health risk.

The traffic police officers working in Turin displayed higher levels of all measured biomarkers, with the exception of cotinine, a biomarker of exposure to tobacco smoke. Overall, these data highlight the influence of the living environment on the biological parameters, showing its influence on both local and general OS and inflammation.

Further investigations could consider a multiple airborne pollutants analysis, also a multi-site sampling for more comprehensive comparisons on the urbanisation levels.

In the future, additional analyses on urban settings, such as greyness/greenness quantification [46], may be helpful in driving new preventive strategies related to urban management, for outdoor workers and the general population.

## Figures and Tables

**Table 1:** Numerical and percentage characteristics of subjects. The statistical differences between the two cities are reported. Age: Mann–Whitney (M–W) U-test, BMI t-test; gender and job duties: X-square test.

		Turin *N* = 109 (71%)	Pavia *N* = 45 (29%)	*p*-Value	Overall *n* = 154
**Age (years)**		45.5 ± 7.7	46.6 ± 7.6	0.390	45.8 ± 7.7
**BMI (kg/m^2^)**		24.8 ± 3.7	24.1 ± 3.2	0.236	24.6 ± 3.6
**Gender**	**Females**	50 (46)	16 (36)	0.153	66 (43%)
	**Males**	59 (54)	29 (64)		88 (57%)
**Job Duties**	**Indoor**	59 (54)	26 (58)	0.765	85 (55%)
	**Outdoor**	50 (46)	19 (42)		69 (45%)

**Table 2:** General description of environmental and biological measurements presented as subgroups by city. The statistical differences between the two cities are reported (M–W U-test).

	Turin	Pavia	*p*-Value	Overall
**FeNO (ppb)**	37.7 ± 28.3	33.6 ± 48.4	<0.001	36.5 ± 35.2
**15-F2t-IsoP (ng/mg of Creatinine)**	9.1 ± 7.3	4.1 ± 2.1	<0.001	7.7 ± 6.7
**FA (µg/m^3^)**	30.9 ± 9.8	17.5 ± 4.7	<0.001	27.0 ± 10.5
**Cotinine (ng/mg of Creatinine)**	15.4 ± 22.8	14.9 ± 28.9	0.641	15.4 ± 23.4
